# Co-creation in health professions education: Triangulating perspectives and processes for deeper insights

**DOI:** 10.3205/zma001796

**Published:** 2026-01-15

**Authors:** Raghdah Al-Bualy, Shireen Suliman, Chloé A. de Mortier, Nicola J. Hancock, Muhammad Zafar Iqbal, Teena Mathew, Jyotsna Sriranga, Astrid Pratidina Susilo, Karen D. Könings

**Affiliations:** 1 Graduate Medical Education, Oman Medical Specialty Board, Muscat, Oman; 2 Maastricht University, School of Health Professions Education, Faculty of Health, Medicine and Life Sciences, Maastricht, The Netherlands; 3 Hamad Medical Corporation, Department of Medical Education, Doha, Qatar; 4 Qatar University, College of Medicine, Doha, Qatar; 5 Maastricht University, CAPHRI, Health Services Research, Maastricht, The Netherlands; 6 Knowledge Institute of Medical Specialists, Utrecht, The Netherlands; 7 University of East Anglia, School of Health Sciences, Norwich, United Kingdom; 8 Acuity Insights, Research Department, Toronto, Ontario, Canada; 9 Urja – Catalysts for Transformation, Karnataka, India; 10 Universitas Surabaya, Faculty of Medicine, Department of Medical Education & Bioethics, Surabaya, Indonesia

**Keywords:** co-creation, triangulation, partnership, dynamics

## Abstract

Co-creation in health professions education (HPE) is gaining momentum as a vital approach to enhancing educational practices and outcomes. As the healthcare landscape evolves, the need for innovative educational models that actively involve learners and other stakeholders in educational design has become increasingly important. This innovative approach promotes learner engagement and ensures that educational programs are more aligned with the realities of contemporary healthcare delivery. Integrating diverse perspectives in co-creation initiatives aims to enrich the educational experience by harnessing the unique insights and experiences of all participants—learners, educators, and healthcare professionals. Though co-creation offers numerous benefits, its success depends on effective partnership dynamics. By considering the insights of participants, moderators, and organizers (also known as triangulation approach), we can identify facilitators, challenges, and opportunities for improvement. Triangulation refers to the process of using multiple sources or methods to gather data and insights. This comprehensive approach will help optimize co-creation initiatives and maximize their benefits for learners and educators alike. This commentary emphasizes the importance of integrating diverse perspectives to enhance our understanding of the co-creation process.

## Abstract (de)

In der Aus- und Weiterbildung in Gesundheitsberufen (Health Professions Education, HPE) gewinnt Co-Kreation zunehmend an Bedeutung als ein zentraler Ansatz zur Verbesserung von Bildungspraktiken und -ergebnissen. Im Zuge der Veränderungen im Gesundheitswesen gewinnt der Bedarf an innovativen Bildungsmodellen an Bedeutung, die Lernende und andere Interessengruppen aktiv in die Gestaltung von Bildungsprozessen einbeziehen. Dieser innovative Ansatz fördert das Engagement der Lernenden und stellt sicher, dass Bildungsprogramme besser mit den Realitäten der heutigen Gesundheitsversorgung übereinstimmen. Die Integration vielfältiger Perspektiven in Co-Kreationsinitiativen zielt darauf ab, die Bildungserfahrung zu bereichern, indem die einzigartigen Einsichten und Erfahrungen aller Beteiligten – Lernender, Lehrender und Gesundheitsfachkräfte – genutzt werden. Obwohl die Co-Kreation zahlreiche Vorteile bietet, hängt ihr Erfolg wesentlich von einer effektiven Partnerschaftsdynamik ab. Durch die Berücksichtigung der Sichtweisen von Teilnehmenden, Moderatoren:innen [aus Gründen der besseren Lesbarkeit wird im Text die Schreibweise mit „:innen“ verwendet. Diese umfasst Personen aller Geschlechter gleichermaßen) und Organisatoren:innen (auch bekannt als Triangulationsansatz) können förderliche Bedingungen, Herausforderungen und Ansatzpunkte zur Optimierung identifiziert werden. Triangulation bezeichnet den Einsatz mehrerer Quellen oder Methoden zur Datenerhebung und Erkenntnisgewinnung. Dieser umfassende Ansatz kann dazu beitragen, Co-Kreationsinitiativen zu optimieren und deren Nutzen für Lernende und Lehrende zu maximieren. Dieser Kommentar unterstreicht die Bedeutung der Integration vielfältiger Perspektiven, um unser Verständnis des Co-Kreationsprozesses zu vertiefen.

## Introduction

Co-creation is gaining momentum in health professions education (HPE) as it promotes diversity, equity, and inclusion, enhances learning experiences, and fosters collaborative relationships between learners and teachers [1]. Co-creation in health professions education (HPE) is a collaborative process that actively engages multiple stakeholders – such as educators, learners, and healthcare professionals – in the design, implementation, and evaluation of educational initiatives to enhance learning outcomes and experiences [1], [2], [3]. In the literature, various terms refer to co-creation, including student-staff partnership, students as partners, co-design, and co-production [4], [5]. Co-creation differs from co-design, which focuses specifically on the design phase, and co-production, which emphasizes the joint creation of services or value, often in the context of healthcare delivery rather than educational design [6], [7]. Co-creation refers to a close collaboration between learners and teachers, aiming to improve teaching and learning by welcoming learners’ perspectives and actively involving them in the educational design process [5]. A typical co-creation initiative includes participants (learners, teachers, and administration representatives), moderators, and organizers. Benefits of co-creation include promotion of learner autonomy, ownership, shared responsibility, self-confidence, and internal motivation, as well as improved quality of educational design [1], [8], [9]. Benefits for teachers include helping them identify learner expectations, learning gaps, and educational needs that contribute to improving teaching practices [10]. Hence, co-creation leads to better educational design, strengthens relationships between educators and learners, and creates a more inclusive learning environment.

Partnership is key to successful co-creation initiatives. Though important, partnerships sometimes fail to manifest. Challenges such as authoritative or resistant behaviors, lack of motivation, unclear expectations and objectives, and poor group dynamics may cause friction between participants and hinder the co-creation process [1], [3], [10]. These challenges may arise from an ineffective co-creation process that does not support true student-staff partnerships. A true partnership occurs when diverse learners actively and directly engage with teachers and invest their time, efforts, and resources to optimize education [2]. Contrarily, co-creation initiatives in which learners are merely passive contributors or explicit attention to partnership dynamics is ignored often result in pseudo-partnership and learners are incorrectly labeled as partners [11], [12]. In such situations, learners are often positioned as sources of data rather than equal partners, and their contributions are limited to providing feedback, completing surveys, or offering quotes used to adapt programs [11], [12]. In other cases, learners are involved in the educational design process but their input is not fully considered in the implementation phase [5], or there is a lack of resolution of the challenges they bring up [13]. Hence, without explicit attention to the co-creation process, these pseudo-partnerships may result in suboptimal educational outcomes, leading to a failure to address the needs of those involved and a wastage of already limited institutional resources.

Co-creation necessitates a reciprocal and equitable process in which all participants contribute actively and equally to various aspects of the educational design experience. These aspects include pedagogical conceptualization, investigation, analysis, decision-making, consensus, and implementation [2]. In recent work, Suliman et al. [12] described key features of true partnership dynamics such as emotions and reactions, sense of familiarity, teachers’ response to students’ ideas, initiation of new ideas, and consensus. These factors collectively highlight that gaining a deeper understanding of co-creation requires explicit attention to the partnership processes. A typical co-creation initiative involves several key players, such as participants (e.g., learners and teachers), moderators (e.g. discussion moderators, observers), and organizers (e.g., research team members, leaders, planners), among others. Each of the above groups plays a specific role in the process and may view manifestation of co-creation differently. Though partnership processes are key in co-creation, less attention has been paid to approaches that lead to successful partnership dynamics [14]. Hence, there is a need for an in-depth exploration of partnership processes from multiple perspectives. This may be achieved through a carefully designed evaluation that considers varied influences (or factors), including meaningful involvement, shared roles, and evaluation methods. The purpose of this commentary is to highlight the importance of integrating diverse perspectives to enhance our understanding of the dynamics involved in the co-creation process.

Through the application of triangulation in evaluation of co-creation initiatives, various perspectives can be explored. Triangulation involves utilizing multiple methods or data sources in qualitative research to comprehensively understand phenomena [15]. Limitations seen in one perspective could be minimized by triangulation with other perspectives. For example, the bias from self-reports could be partly minimized by considering the moderator’s perspective. In the following section, we draw from the literature and outline the co-creation process from various perspectives: through the participants’ eyes, the moderators’ eyes, and the organizers’ eyes. 

## Viewing co-creation through the participants’ eyes

Participant views are crucial to understanding those factors that best facilitate true co-creation including the impact of power dynamics within the group, and ways to mitigate them [13]. Exploring participant perspectives through individual reflection (i.e., via questionnaires or interviews) provides valuable information. An example of a quantitative approach is the work of De Hei et al. [16], who created a validated questionnaire to capture various perspectives and lived experiences of individual participants, allowing for a comprehensive evaluation of the co-creation process. Both questionnaires and interviews can be used to explore participants’ views on shared responsibility, accountability, and motivation [16], [17], and how their prior experiences (educational level, co-creation experience, topic familiarity) might have influenced their perspectives [18]. It is important to explore whether participants felt safe while sharing opinions and stepping outside their comfort zones. Psychological safety is a necessity to ensure that participants voice their views openly without hesitation [19], [20]. 

Participant perspectives could be different from the viewpoints of moderators or organizers. Sometimes participants may have felt uncomfortable sharing their viewpoints while others may view their participation as taking initiative [21]. Therefore, despite the value of eliciting participants' perspectives on the co-creation process, this potential discrepancy necessitates a more holistic exploration to better inform the co-creation process.

## Viewing co-creation through the moderators’ eyes

In their recent work, Suliman et al. [19] recommended capturing feelings and emotions from a neutral view to better understand group dynamics. This neutral view may be accomplished through the perspectives of moderators or observers. 

The moderator influences the process by encouraging participation and asking stimulating questions. Their dual role of coordinating the discussion and observing the dynamics can make it challenging to assess if true co-creation process has taken place [22]. Therefore, inviting an observer can be valuable. Observers are focused solely on evaluating the process and their influence on the co-creation dynamics is minimal [22]. They are focused on gathering information on group dynamics and participant interactions during the sessions, with limited input and interaction with participants to allow participants to co-create freely. 

The moderator's role should be determined by the research paradigm that underpins the aim(s) of exploration. For instance, a post-positivist approach might favor a pure observer to assess the "true" nature of co-creation, while a critical theory approach might benefit from a moderator who supports and advocates for the process [23]. Regardless of their role, reflexivity is essential for moderators and observers to understand their own biases and how they might influence the co-creation process [24]. 

## Viewing co-creation through the organizers’ eyes

Organizers may include leaders, planners of co-creation initiatives, and/or research team members. Organizers usually have a holistic view of the overall project and therefore, they play a key role in assessing the alignment between project goals and outcomes achieved [25]. Their role starts before the initiation of the project by ensuring all stakeholders, particularly those from traditionally underrepresented groups, are invited to participate. During analysis of the sessions, through reading co-creation session transcripts, organizers may assess whether all stakeholders were actively engaged and whether their contributions were valued. Organizers may eventually have a role in measuring co-creation sustainability and long-term impact [26]. 

As with others, this group needs to be aware of their inherent biases and how these might influence the design and analysis of the process. Potential biases may include a bias toward valuing traditional forms of expertise over student or community input, leading to an underappreciation of the diversity of contributions [25]. Organizers may also exhibit confirmation bias by focusing their analysis on outcomes that align with their preconceived expectations or the original project goals, potentially overlooking unexpected but valuable innovations that emerged during the co-creation process [27]. Organizers should adopt reflexive practices, such as actively seeking diverse perspectives and engaging in critical self-assessment throughout the project [2].

## Conclusion

Co-creation offers a promising approach to educational design in HPE. To fully unlock its potential, understanding the co-creation process should be an integral component of evaluating and optimizing it. In this commentary, we provide a brief overview of how capturing different stakeholders’ perspectives is beneficial to successful outcomes. Future research focusing on the evaluation of co-creation initiatives should consider the triangulation of perspectives of all involved in the co-creation process, including participants, moderators, and organizers. 

## Authors’ ORCIDs

- Raghdah Al-Bualy: [0000-0003-3676-0282]
- Shireen Suliman: [0000-0003-0319-5109]
- Chloé A. de Mortier: [0000-0002-5911-7698]
- Nicola J. Hancock: [0000-0003-4850-3152]
- Muhammad Zafar Iqbal: [0000-0002-5605-8143]
- Teena Mathew: [0009-0007-3754-385X]
- Jyostna Sriranga: [0000-0001-7850-5664]
- Astrid Pratidina Susilo: [0000-0002-4371-1721]
- Karen D. Könings: [0000-0003-0063-8218]

## Acknowledgements

The authors would like to express their sincere thanks to Dr. Stefanie Mosimann MHPE, University Hospital Bern, Switzerland, for her linguistic support in translating the English text.

## Competing interests

The authors declare that they have no competing interests. 
